# Obesity in Older Type 2 Diabetic Patients: Does Working Environment Add Vulnerability?

**DOI:** 10.3390/ijerph15122677

**Published:** 2018-11-28

**Authors:** Maria Piedade Brandão, Margarida Fonseca Cardoso

**Affiliations:** 1 ESSUA—Health School, University of Aveiro, 3810-193 Aveiro, Portugal; 2 CINTESIS—Center for Health Technology and Services Research, University of Porto, 4200-450 Porto, Portugal; 3 ICBAS—Instituto de Ciências Biomédicas de Abel Salazar, University of Porto, 4050-313 Porto, Portugal; mcard@icbas.up.pt

**Keywords:** ageing, employment, Europe, obesity, type 2 diabetes

## Abstract

Little is known about how working adults with type 2 diabetes are managing their health. This study aims to analyze the associations between health, behavioral, and sociodemographic characteristics and obesity in older diabetic patients in Europe. Data from the Survey of Health, Ageing, and Retirement in Europe was used to compare 1447 participants that were identified as having type 2 diabetes with 28,047 participants without diabetes. Multilevel logistic models stratified by type 2 diabetes examined the relationships of health, behavioral, and sociodemographic characteristics with obesity. The proportion of physical inactivity was significantly higher among those with type 2 diabetes (15.0% vs. 6.1%). Individuals with diabetes had more chronic diseases, more limitations in activities, higher body mass index, more depression, lower quality of life and well-being, and lower employment rate. Among those with type 2 diabetes, those employed were more likely to be obese (OR = 1.377, 95% CI, 1.023 to 1.853) and women were 52% more likely to be obese than men. The surveillance of weight in working environments should be required within workers with type 2 diabetes. It is concluded that this and other adjustments could be beneficial in people with diabetes.

## 1. Introduction

Currently, type 2 diabetes is considered to be an epidemic disease [1] and is one of the major causes of mortality from non-communicable diseases [2]. Most of the subjects with type 2 diabetes are overweight or obese, which implies the constant need to draw attention to this problem. In recent years, several studies have been conducted in different countries with various approaches and research designs that show the association between type 2 diabetes and obesity [3,4]. They also show that when obesity is present in diabetics there are limitations in activities of daily living and physical activities [2,3,5,6]. Diabetes is known to have a negative impact in employment, leading to an early exit from the workforce [7]. Absence of employment on older age has been found to have negative effects on psychological well-being [8] and cognitive ability [9], however an unhealthy environment at work can lead to early retirement [8,10]. Diabetes was recognized as one of the main factors associated with premature losses in the workforce and among those who are employed and have diabetes, working in a strenuous environment considerably increases the risk of early exit [7].

Scientific evidence has shown that genetic and environmental factors contribute to the onset of the disease and that diabetics are 15% more likely to die prematurely [11]. It is also known that the genetic factor has been associated with this pathology, however it was only reflected in 10% of the cases [12]. When the genetic component exists, there is an increased risk of type 2 diabetes in people who are simultaneously exposed to non-healthy lifestyles, such as smoking, physical inactivity, and fat and/or sugar intake [13].

Despite the awareness, promotion, and prevention campaigns proposed by many researches and stakeholders that have contributed to diminish the detrimental impact of type 2 diabetes [14,15], this disease’s prevalence has increased in Europe [2,16]. In 2013, the prevalence of diabetes was estimated as varying between 2.4% in Moldova and 14.9% in Turkey [2]. The role of the diabetes self-management education is well known in the improvement, prevention, and care of diabetes through the empowerment of people, mainly those with low health literacy levels. A recent study showed that the existence of diabetes self-management education varied between European countries, with some countries reporting several programs and others reporting none [17]. In the same vein, other scientific evidence show that supportive environments help employees to manage their disease, resulting in the prevention of absenteeism and loss of productivity [18]. However, there is still a lack of support at the workplace from employers and managers on the implications of diabetes on their employees.

In light of a growing number of surveys worldwide, the Survey of Health, Ageing, and Retirement in Europe (SHARE) appears as an important database in Europe that allows for the assessment of the profile of people with type 2 diabetes after being diagnosed. SHARE is a multinational survey, which involves a large number of cohorts from all over Europe. The SHARE target population consists of six panel waves (2004, 2006, 2010, 2012, 2014, and 2017). The last wave (Wave 6) consists of respondents over 50 years old from 18 countries: Austria, Germany, Sweden, Spain, Italy, France, Denmark, Greece, Switzerland, Belgium, Israel, Czech Republic, Poland, Luxembourg, Portugal, Slovenia, Estonia, and Croatia.

The present study was undertaken to analyze the association between obesity and sociodemographic, behavioral and health factors in European older adults with type 2 diabetes. Differences in the direction and strength of the associations with obesity were explored between people with and without type 2 diabetes, including the associations between being employed and obesity. This study contributes towards filling the gap of much-needed data to understand how working adults with type 2 diabetes across Europe are managing their health towards a better state of physical and general well-being.

## 2. Materials and Methods

### 2.1. Data and Measures

A cross-sectional analysis was performed on the basis of the results from survey SHARE’s Wave 6 (2017: baseline) as secondary analysis of existing data [19], because SHARE’s project researchers share the cleaned data with the authors. SHARE is the Survey of Health, Aging, and Retirement in Europe and includes data on health, socio-economics and social and family network of Europeans aged above 50 years old. SHARE was founded in 2002 and it currently has six waves of data collection that are available on the project’s website (http://www.share-project.org). The website also has detailed methodological information available for researchers. In this study we used the most recent wave of SHARE, wave 6 (the field work was completed in November 2015 and released in March 2017) [20,21] that includes data from 18 countries. The authors are registered users of the SHARE project and are allowed to use the data for scientific purposes. More information about the SHARE-project is available on http://www.share-project.org. The methodological steps were closely detailed elsewhere [20].

In this study, men and women aged between 51 and 74 years old from 18 European countries were included. Participants older than 74 years old were not included since almost all were not currently employed. Participants were considered to have diabetes if answered positively to one of the two following questions: (1) “Has a doctor ever told you that you had any of the conditions on this card? Diabetes or high blood sugar” or (2) “Do you currently take drugs at least once a week for problems mentioned on this card? Drugs for diabetes”. Participants were classified has having type 1 diabetes if the age when diabetes was diagnosed was under 20 years old, and they were classified as having type 2 diabetes if diagnosis was when they had at least 20 years old [22].

Throughout the study, participants provided personal and general information that were used in this analysis: gender (male or female), age (65 to 74 vs. 51 to 64) marital status (married/registered partnership or other), employment status (employed or other), and education level. The possible answers for the current job situation were retired, employed or self-employed, unemployed, permanently sick or disabled, home-maker, or other. Participants were classified as employed if they were employed or self-employed. The education level was collected according to the International Standard Classification of Education (ISCED-11) [23] and was analyzed in this study as upper secondary or more (ISCED ≥ 3) vs. lower secondary education or less (ISCED < 3). Health behaviors included: smoking habits (current smoker or other), physical inactivity, and hazardous drinking. Current smoker was considered when the participants reported smoking at the present time. Physical inactivity was defined as never or almost never engaging in neither moderate nor vigorous physical activity. Hazardous drinking was considered when the participants reported that the frequency of six or more drinks per three or four days a week or more in the last three months.

The subjective conditions of wellbeing of participants in this study were collected through the self-rated healthy variable (yes or no), the 12-item Control, Autonomy, Self-realisation, Pleasure (CASP-12) scale to measure the quality of life [24,25], and the EURO-D scale for depressive mood [26]. The global measure of self-perception of health uses a five-point scale with response options “excellent”, “very good”, “good”, “fair”, and “poor”. The first three response options were collated in the category ‘self-rated healthy’ and the other two options in the category ‘self-rated unhealthy’. Self-perception of health by older people has been shown to have a predictive power in various events, such as hospitalization, mortality, and functional decline [27]. The quality of life was measured using the CASP-12 scale corresponding to the sum of 12 Likert-scaled items. Total scores ranged 12–48 with higher scores indicating better quality of life [20,28]. The EURO-D scale has 12 items (depressed mood, pessimism, wishing death, guilt, sleep, interest, irritability, appetite, fatigue, concentration, enjoyment, and tearfulness) and each item is scored 1 or 0 (respectively if the symptom is present or not). Within SHARE, a score greater than 3 from all items indicates depression [26,29]. Also the presence of chronic diseases, limitations with activities of daily living, and obesity were analyzed as a binary variable: if participants had two or more chronic diseases, if participants had one or more limitations with activities of daily living, and if their body mass index was 30 or above. Having two or more chronic diseases and one or more limitations with activities of daily living were variables created and provided within the SHARE database based on a range of specific diagnoses and symptoms, as well as difficulties with a range of (instrumental) activities of daily living reported by the participants.

The SHARE was reviewed and approved by the Ethics Committee of the University of Mannheim until July 2011. Since 2011, the Ethics Council of the Max Planck Society for the Advancement of Science (MPG) is responsible for all requisite ethical considerations. More details about the ethics procedure can be found in http://www.share-project.org/organisation/dates-facts.html.

This research was guided by international research ethics principles, such as the Respect Code of Practice for Socio-Economic Research and the Declaration of Helsinki.

### 2.2. Analysis

Descriptive statistics were used to describe the characteristics of those without diabetes and those identified has having type 2 diabetes. Categorical variables were described as proportions and compared using chi-square test (with Yates’s continuity correction). Quantitative variables were described as mean ± standard deviation and compared with the independent sample t-test.

Associations of the different variables with obesity were investigated using multilevel logistic regression with individuals nested in countries. As a first step, univariable associations between obesity and each variable were investigated. As a second step, the association of each variable with obesity was obtained by controlling all others in the multivariable model, and non-significant factors (*p* > 0.05) were dropped from the final model based on a stepwise procedure. We started with the full model, with all independent variables in the model, and then deleted them one by one until all variables were significant. Variables where the majority of the values were missing were not considered in the multivariable analysis. As a third step, the interactions between diabetes and each variable were investigated. Unadjusted and adjusted odds ratio (OR and AOR) and 95% Confidence Intervals (CI) were estimated.

All statistical tests were two-tailed and a *p* value lower than 0.05 was considered to be statistically significant. Statistical analyses of the data were performed with SPSS, version 24.0 (IBM, Armonk, New York, NY, USA).

## 3. Results

From a total of 32,418 participants aged between 51 and 74 years old, 13.6% were diagnosed with diabetes (*n* = 4371), the age when diabetes was diagnosed was available for a total of 1469 participants, and 1447 (98.5%) were identified has having type 2 diabetes. The analyzed sample comprises 1447 participants with a diagnostic of type 2 diabetes and 28,047 participants without a diabetes diagnosis.

### 3.1. Socio-Economic Variables

Table 1 shows the sociodemographic characteristics of those with type 2 diabetes as compared with those without diabetes across the two age groups: 50 to 64 and 65 to 74 years old. The proportion of females (around 60%) and the marital status were comparable between those with type 2 diabetes and without diabetes for the two age groups. The proportion of those employed was significantly lower among those with diabetes (*p* < 0.001). Although a sharp decrease in employment is observed from the younger to the older, in both age groups the proportion of those employed among those with type 2 diabetes was about half of those without diabetes.

A significantly lower percentage (*p* < 0.001) with upper secondary education or more was found among those with type 2 diabetes. The proportion with upper secondary education or more decreased with age, with a gap of around 20% between those with type 2 diabetes when compared with those without diabetes. The information about marital status and education was only available for a small fraction of the participants, 25.6% and 43.4%, respectively.

### 3.2. Behavioural Risks Variables

The smoking status was only available for 20.7% of the participants. The proportion of current smokers was very similar between participants with type 2 diabetes and participants without diabetes (Table 1) The majority of the participants were physically active, however a significantly increased proportion of physical inactivity was found among those with type 2 diabetes (15.0% vs. 6.1%). Less than 4% of the participants reported hazardous drinking, irrespective of having been diagnosed with diabetes.

### 3.3. Health Related Variables

Those identified as having type 2 diabetes self-rated their health less often as healthy (Table 1), had lower levels of quality of life, and had a higher proportion of depressive caseness. Furthermore, an increased proportion of persons with two or more chronic diseases (85.0% vs. 37.1%), one or more limitations with activities of daily living (17.4% vs. 6.5%), and obesity (47.9% vs. 20.5%) were found among those with type 2 diabetes.

### 3.4. Multivariable Analysis of Obesity

In the univariable analysis, obesity was associated significantly with having type 2 diabetes, being older, being a woman, and not being employed. It was also associated with having a lower education level, not being a smoker, physical inactivity, not perceiving their health as healthy, having lower levels of quality of life, having limitations in activities of daily living, and having two or more chronic diseases (Table 2).

Multivariable analysis was considered to analyze the associations of sociodemographic, behavioral risks and health characteristics with obesity, adjusted for all other variables. Being married, having upper secondary education or more, and being a current smoker were not considered in the multivariable analysis because most of the participants did not have information on those variables. After adjusting for the effects of the other variables, multivariable analysis showed that only type 2 diabetes, age, gender, employment, self-perceived health, physical inactivity, limitations with activities of daily living, and chronic diseases were associated independently and significantly with obesity (Table 2). Hazardous drinking, depressive caseness, and CASP index for quality of life and well-being were excluded from the equation. Two significant interactions were found, an interaction between diabetes and gender, and an interaction between diabetes and employment, implying that the associations between obesity and gender, and obesity and employment, differed between those with type 2 diabetes from those without diabetes. For both with type 2 diabetes and without diabetes, the odds of being obese decreased with age and increased between 25% and 65% with physical inactivity, limitations with activities of daily living, and chronic diseases. Those who self-rated healthy were less likely to be obese. Among those without diabetes, gender was not associated with obesity, and those employed were less likely to be obese (AOR = 0.883, 95% CI, 0.818 to 0.953). While among those with diabetes type 2, women were 52% more likely to be obese than men and those employed were more likely to be obese (AOR = 1.377, 95% CI, 1.023 to 1.853).

## 4. Discussion

This present study provides novel information on the association between employment (being currently employed or self-employed vs. others) and obesity. It is well known that obesity is the major risk factor for type 2 diabetes. While those who did not have diabetes had a protective effect for obesity because of employment (AOR = 0.833), those with type 2 diabetes had increased odds of being obese (AOR = 1.377). Females were more likely than men to have obesity among those with type 2 diabetes (AOR = 1.522). According to some researchers, there are more females with obesity than males, mainly after the age of 45 years [30]. This evidence is, on the one hand, explained by income inequalities [31], and, on the other hand, explained by the associations between socio-economic measures and obesity that differs between men and women [31,32,33].

In this study, the estimated proportion of people with diabetes between 51 and 74 years old in Europe was 13.6%, in which 98.5% of them had type 2 diabetes. The combination of these percentages resulted in a proportion of 13.3% of the people under the designated age group having type 2 diabetes. In 2013, a 8.5% prevalence of type 2 diabetes in Europe was estimated, corresponding to a population of 20 to 79 years old. Although our estimated proportion was higher, our study considered individuals between 51 and 74 years old. Realising that the older the person is, the greater is the risk of developing diabetes, an increase of individuals identified as having type 2 diabetes was to be expected. The prevalence of diabetes in Europe is also increasing among people of all ages [2,34].

When compared to individuals who did not have diabetes, those with type 2 diabetes had lower education levels, lower rate of employment, were less physically active, had more limitations of daily living, more chronic diseases, and were more obese. Since obesity is one of the most important risk factors for the development of type 2 diabetes and among people already with type 2 diabetes, one of the risk factors for the development of complications, a deeper analysis of obesity was performed. Almost half (47.9%) of our analyzed sample of Europeans with type 2 diabetes aged between 51 and 74 years old, were obese. Obesity was found to be associated significantly and independently with type 2 diabetes, age, gender, employment, physical inactivity, self-perceptions of healthy, having one or more limitations with activities of daily living, and having two or more chronic diseases. Both individuals with or without type 2 diabetes when being physical inactive, having limitations with activities of daily living and chronic diseases were associated with an increased odds of being obese, while the self-perception of being healthy was not. Only among people with type 2 diabetes, being female was significantly associated with obesity, making being female an even greater vulnerable subgroup. Although weight is already a concern for all of those with type 2 diabetes, special recommendations should be designed for female patients.

Several previous studies reported that physical inactivity might be a significant factor in the increase of diabetes, therefore, increased physical activity should contribute to the management of type 2 diabetes and it may even reduce the risk of diabetes [35]. Managing diabetes is a seemingly easy task but it is difficult to get around. The tasks that are considered to be the most important for the prevention and management of type 2 diabetes are the control of weight, intake of fats and sugars, as well as increased physical activity [35]. However, data from the WHO report in 2016 reinforces the difficulty of type 2 diabetes management because it refers that in Europe about 60% of people with type 2 diabetes are obese [34].

Simple implementations, such as low-calorie diets, have been shown to be very beneficial (≤800 kcal/day), and may even contribute to the remission of diabetes, as tested in a very recent longitudinal prospective study [36]. Given the lack of physical activity in the life style of people, physicians may have to consider having low-calorie diets (e.g., carbohydrate restrictions) in an elderly’s life, especially among diabetics [3,37,38]. Individuals who are overweight or obese have a significantly increased risk of developing diabetes, a risk that is about three times higher than the population considered to have a normal weight [4].

Scientific evidence has shown that smoking increases the risk of being diabetic [39] and also appears to influence fat distribution patterns [40,41]. The latter evidence is very important, since it is known that central obesity (association of waist-hip ratio and waist circumference) is a risk factor for insulin resistance and men who smoke tend to have higher accumulation of abdominal fat than nonsmokers [42,43]. Both diabetes and smoking are known to be associated with an increased risk of developing non-communicable diseases, such as the cardiovascular diseases, so diabetics who smoke are a very vulnerable subgroup. However, in the analyzed sample, irrespectively of having type 2 diabetes or not, the proportion of current smokers was around 30% among those who aged 51 to 64, and 15% among those aged 65 to 74 years old. Smoking cessation should be part of the healthy life styles recommended for people with diabetes, and, as a result, the prevalence of smoking should have a significant decrease.

Healthy lifestyles are recommended to people with type 2 diabetes, and this will surely have a positive impact on their health status [2,3,5,6]. However, our study showed that the well-being (depressive caseness and quality of life) and self-perception of health were lower among those with type 2 diabetes when compared to people without diabetes.

Although the mechanisms underlying the relationship between employment and diabetes remain unclear, diabetes has been recognized on having a prominent role in premature losses to the work force [10]. Our results show that, among those with type 2 diabetes, the proportion that was employed was about half when compared to people without diabetes. People with type 2 diabetes had a higher proportion of unfavorable health status (more chronic diseases, more limitations, depression, and lower quality of life and well-being) and these could be the reasons for the reduced rate of employment.

When associating employment with obesity, opposite trends were found for people with type 2 diabetes and people without diabetes. For people without diabetes, not having employment was associated with increased odds of being obese, while for people with type 2 diabetes, not having employment seems to have a protective effect. Maybe employment limits the necessary time required for the adoption of healthy life styles recommended for people with diabetes. A large study with older adults in a representative sample in Korea showed that unemployment is significantly related to a higher risk of obesity, regardless of age, lifestyle, and socioeconomic factors [44]. Nonetheless, the positive association that was found in our study between employment and obesity among diabetics can be related with the difficulty of diabetics in decreasing their excess weight while having fixed work schedules. The adoption of healthy life styles includes physical activity and a daily diet with calorie restrictions, something that is particularly important for people with diabetes [45]. However, in order to have an effect on the loss of weight, healthy life styles should be adopted on a regular basis. In addition, the surveillance of weight in the employed group with type 2 diabetes should be required in order to control for diabetes in the working environments. This is a highly complex process that requires time, health education, and, in particular, the willingness of employers to enforce measures to help diabetics manage and control their diabetes. Thus, a reduction of the working hours and health promotion in the workplace should be considered for those with diabetes. Research has shown that health promotion in the workplace could contribute to changes in health behavior (namely dietary behaviors [46] and physical activity [47]), resulting in an impact in the reduction of obesity [48,49]. An early exit from the workforce might have negative impacts on the psychological well-being [8], cognitive functions [9], health, and financial status [10], so it should be avoided. The adverse effects on individuals and their families, as well as on society, on health, and social protection systems, highlight the benefits that good working conditions can give in keeping older individuals in the workforce [7,8,10].

### Strengths and Limitations

This paper has a few strengths including the use of cross-national comparable data. Previous studies did not often consider the reflex of simple measures, like diets or physical activity on managing diabetes, despite being more appropriate indicators, as compared to more complex measures, such as pharmacologic management [50]. Behavioral differences in people with type 2 diabetes and without diabetes were also explored, while previous studies have often neglected them [51]. The associations between being employed and obesity were compared between people with or without diabetes, and not just the rates of employment.

However, this study has some limitations that should be acknowledged. This work was based on cross-sectional data from a public survey, which makes it impossible to infer causal relations, and is only possible to establish in experimental or longitudinal studies. The design used does not provide data on diabetics’ behaviors over time. In addition, the cross-sectional design of the study does not allow acknowledge whether employment protected the development of diabetes or if the diagnosis of diabetes was the reason for not being employed in the first place.

The estimated proportions of type 2 diabetes should be read with caution, since the categorization of type 2 diabetes was possible only in one-third of those identified with the disease. The National Diabetes Statistics Report from the Centers for Disease Control and Prevention refers type 2 diabetes as being responsible for 90–95% of all diabetes cases in U.S. [52], similar to the 98.5% found in the analyzed European sample. A participant that was diagnosed with diabetes before the age of 20 could have been misclassified has having type 1 diabetes instead of type 2 diabetes. However, type 2 diabetes was considered to be rare in youngsters, before the 1990s, when an increase of the incidence and prevalence had begun [53]. In addition, the variable that represents the group with type 2 diabetes, in the SHARE database, was collected in a way that involves the answers to three different questions by the participants. Since it was not possible to confirm the diagnosis of diabetes with a second information source, a possible selection bias could have been introduced into our study. However, the reporting bias of diabetes is considered negligible, and therefore health survey data is a valuable way to study this condition [22].

## 5. Conclusions

In conclusion, our study shows that, when compared to the general population, participants with type 2 diabetes had lower education levels, lower employment rates, were less physically active, had a higher number of limitations of daily living, had more chronic diseases, and were more obese. Not being employed seems to have a protective effect for those with type 2 diabetes, because it reduces their likelihood of being obese. Among those with type 2 diabetes, women were more likely to be obese than men. Our results highlight the need for the improvement of working conditions, which will help older diabetic workers to stay employed. The adjustment of the working environment on a personal sense, while considering the individual’s health condition, will prevent an early exit from the workforce and it contributes to a successful aging, and as a result, both the individual and society will benefit.

## Figures and Tables

**Table 1:** Comparison of socio-demographic, behavioral risks and health characteristics between participants with type 2 diabetes and without diabetes for the two age groups, between 51 and 64 and between 65 and 74 years old.

	Age Group	With Type 2 Diabetes		Without Diabetes	
Age	51–64	N = 660		N = 16,349	
	65–74	N = 787		N = 11,698	
	Total	N = 1447		N = 28,047	
		*n*	%	*n*	%
Female	51–64 **	362	54.8%	9842	60.2%
	65–74	455	57.8%	6948	59.4%
	Total	817	56.5%	16,790	59.9%
Married	51–64 *	225	71.4%	3234	65.0%
	65–74	173	60.1%	1141	57.5%
	Total	398	66.0%	4375	62.9%
Employed	51–64 ***	211	32.1%	9026	55.5%
	65–74 ***	23	3.0%	751	6.5%
	Tota l ***	234	16.4%	9777	35.1%
Upper secondary or more	51–64 ***	152	52.8%	5999	70.7%
	65–74 ***	99	37.8%	2259	59.8%
	Total ***	251	45.6%	8258	67.3%
Current smoker	51–64	78	26.9%	1310	31.0%
	65–74	37	14.1%	204	15.3%
	Total	115	20.8%	1514	27.3%
Physical inactivity	51–64 ***	83	12.6%	877	5.4%
	65–74 ***	134	17.1%	827	7.1%
	Total ***	217	15.0%	1704	6.1%
Hazardous drinking	51–64	27	4.1%	589	3.6%
	65–74	20	2.5%	421	3.6%
	Total	47	3.3%	1010	3.6%
Self-rated healthy	51–64 ***	262	39.7%	11,967	73.3%
	65–74 ***	268	34.1%	7686	65.8%
	Total ***	530	36.7%	19,653	70.2%
Depressive caseness	51–64 ***	250	38.6%	4027	25.0%
	65–74 ***	298	39.0%	2736	23.7%
	Total ***	548	38.8%	6763	24.5%
CASP index for quality of	51–64 ***	35.35	±6.40	37.90	±6.02
life and well-being	65–74 ***	34.71	±6.66	37.81	±6.13
Mean ± SD	Total ***	35.01	±6.55	37.86	±6.07
1 + limitations with activities	51–64 ***	101	15.3%	870	5.3%
of daily living	65–74 ***	150	19.1%	936	8.0%
	Total ***	251	17.4%	1806	6.5%
2 + chronic diseases	51–64 ***	532	80.6%	5032	30.8%
	65–74 ***	696	88.7%	5349	45.8%
	Total ***	1228	85.0%	10,381	37.1%
Obese	51–64 ***	329	50.4%	3242	20.1%
	65–74 ***	354	45.8%	2410	21.0%
	Total ***	683	47.9%	5652	20.5%

* *p* < 0.05, ** *p* < 0.01, *** *p* < 0.001 for the comparison of type 2 diabetic group vs. group without diabetes, for each age group; Missing values: Married (74.4%), Upper secondary or more (56.6%), Current smoker (79.3%), all other variables (<2%); CASP, 12-item Control, Autonomy, Self-realisation, Pleasure scale; SD, Standard Deviation.

**Table 2:** Associations of sociodemographic, behavioral risks and health characteristics variables with obesity from multilevel (random intercept) univariable and multivariable logistic regression with individuals nested in countries (Odds Ratios < 1 are associated with decreased probability of obesity).

	Univariable Analysis		Multivariable Analysis	
	Odds Ratio	95% CI	Adjusted Odds Ratio	95% CI
Type 2 diabetes	3.230 ***	2.891 to 3.608	a	
Age 65 to 74	1.070 *	1.010 to 1.133	0.868 ***	0.811 to 0.929
Female	1.085 **	1.024 to 1.150	a	
Among those without diabetes			1.015	0.954 to 1.080
Among those with diabetes			1.522 ***	1.223 to 1.894
Married	1.026	0.914 to 1.151		
Employed	0.755 ***	0.709 to 0.804	a	
Among those without diabetes			0.883 **	0.818 to 0.953
Among those with diabetes			1.377 *	1.023 to 1.853
Upper secondary or more	0.658 ***	0.596 to 0.726		
Current smoker	0.609 ***	0.526 to 0.705		
Physical inactivity	1.879 ***	1.692 to 2.087	1.257 ***	1.121 to 1.410
Hazardous drinking	0.966	0.828 to 1.126		
Self-rated healthy	0.509 ***	0.479 to 0.540	0.714 ***	0.667 to 0.764
Depressive caseness	1.401 ***	1.315 to 1.493		
CASP index for quality of				
life and well-being	0.964 ***	0.959 to 0.969		
1 + limitations with activities of daily living	2.296 ***	2.085 to 2.528	1.511 ***	1.359 to 1.681
2 + chronic diseases	2.109 ***	1.992 to 2.234	1.646 ***	1.544 to 1.755

* *p* < 0.05, ** *p* < 0.01, *** *p* < 0.001, 95% CI: 95% confidence interval; ^a^ significant interactions: type 2 diabetes x Female, type 2 diabetes x Employed; Missing values: Married (74.4%), Upper secondary or more (56.6%), Current smoker (79.3%), all other variables (<2%); CASP, 12-item Control, Autonomy, Self-realisation, Pleasure scale.
